# Adherence to Dietary Recommendations for Red and Processed Meat in Poland: Insights from the 2017–2020 National Nutrition Survey

**DOI:** 10.3390/nu17050790

**Published:** 2025-02-25

**Authors:** Alicja Kucharska, Beata Irena Sińska, Mariusz Panczyk, Piotr Samel-Kowalik, Dorota Szostak-Węgierek, Filip Raciborski, Bolesław Samoliński, Iwona Traczyk

**Affiliations:** 1 Department of Human Nutrition, Faculty of Health Sciences, Medical University of Warsaw, 01-445 Warsaw, Poland; beata.sinska@wum.edu.pl; 2 Department of Education and Research in Health Sciences, Faculty of Health Sciences, Medical University of Warsaw, 00-581 Warsaw, Poland; mariusz.panczyk@wum.edu.pl; 3 Department of Prevention of Environmental Hazards, Allergology and Immunology, Faculty of Health Sciences, Medical University of Warsaw, 02-007 Warsaw, Poland; piotr.samel-kowalik@wum.edu.pl (P.S.-K.); filip.raciborski@wum.edu.pl (F.R.); boleslaw.samolinski@wum.edu.pl (B.S.); 4 Department of Clinical Dietetics, Faculty of Health Sciences, Medical University of Warsaw, 01-445 Warsaw, Poland; dorota.szostak-wegierek@wum.edu.pl; 5 Department of Public Health, Faculty of Health Sciences, Medical University of Warsaw, 02-106 Warsaw, Poland; iwona.traczyk@wum.edu.pl

**Keywords:** red meat, processed meat, intake, adherence to dietary recommendations, Polish population

## Abstract

**Background/Objectives:** Meat is an important source of nutrients, but the excessive consumption of red and processed meat raises concerns related to its association with chronic diseases. This study aimed to analyze the consumption of red and processed meat in Poland, compare it to dietary recommendations and examine sociodemographic factors affecting consumption patterns. **Methods:** Data from two representative studies conducted in 2017–2020, including 4000 adult inhabitants of Poland, were analyzed using a repeated 24-h recall to estimate the intake of red meat (RM), processed meat (PM), and combined red and processed meat (CRPM). CRPM intake was compared to national recommendations. Statistical analyses were performed using weighting to improve result generalization and adjust for demographic distribution errors. **Results:** The mean daily intake of CRPM was 139.0 g, including 64.0 g of RM and 75.3 g of PM. PM accounted for 59.4% of CRPM intake. Men consumed significantly more CRPM than women (171 g/day vs. 106 g/day, *p* < 0.001). Significant differences were found between age groups in RM consumption (*p* < 0.001) with younger individuals consuming more RM. PM intake was highest among middle-aged respondents (35–54 years, *p* < 0.001). Consumer profiles most closely aligned with the recommended ≤70 g/day intake included women (OR = 0.36, *p* < 0.001), older individuals (OR = 1.01, *p* = 0.002), and those with higher education (OR = 1.38, *p* = 0.010). **Conclusions:** The results indicate a significant exceedance of recommended meat intake, particularly processed meat, posing a health risk. Targeted public health interventions are needed, especially for younger men and middle-aged groups, to reduce processed meat consumption and promote healthier alternatives.

## 1. Introduction

Adherence to dietary recommendations for red and processed meat consumption is a critical factor in reducing the health risks associated with excessive meat consumption. The impact of meat consumption on health depends on its type, amount and the general nutritional pattern [1,2,3]. While white meat is generally considered to be neutral or beneficial for health [4,5,6,7,8], the excessive consumption of red and processed meat raises serious concerns about the increased risk of non-communicable chronic diseases.

In line with the classification of the World Health Organization (WHO) and the International Agency for Research on Cancer (IARC) the term “red meat” refers to fresh unprocessed mammalian muscle meat, including beef, veal, pork, lamb, mutton, horse and goat meat, both minced and frozen [9]. In contrast “processed meat” includes meat products preserved by salting, smoking, drying, fermentation, or the addition of chemical preservatives, such as ham, bacon, salami, sausages and canned meat, mainly from pork or beef, but also from other meats, poultry and offal [10].

Numerous studies showed that high consumption of red and processed meat increased the risk of all-cause mortality, cardiovascular diseases, type 2 diabetes, hypertension, and certain types of cancer, such as colorectal, pancreatic, and liver cancer [11,12,13,14,15,16,17,18]. Red meat was classified as probably carcinogenic and processed meat as carcinogenic to humans [9]. The negative impact of meat on health results both from the presence of natural ingredients, such as saturated fatty acids and heme iron, as well as ingredients or chemicals added or formed during processing that promote the formation of carcinogens and pro-inflammatory compounds [10]. Additionally, a diet rich in high-calorie meat products often leads to obesity, contributing to excessive energy intake [19].

To address these risks, international and national dietary recommendations advocate for limiting the consumption of red and processed meat. Current dietary recommendations suggest that adults should consume between 98 g and 500 g of red meat per week, with processed meat intake restricted to a maximum of 70 g per day [1]. The EAT-Lancet Commission sets the most stringent limits, recommending just 98 g of red meat per week while advising to minimize or completely exclude processed meat [20]. In most European countries, including Poland, the recommendations for the consumption of red and processed meat are up to 500 g per week, although in Belgium and Slovenia they are more restrictive (300 g per week) [21,22]. In France and Belgium, processed meat limits are 150 g and 30 g per week, respectively [21,23]. The European Society of Cardiology (ESC) recommends limiting red meat consumption to 350–500 g per week and limiting processed meat consumption to a minimum [24], indicating a scientific consensus on this issue.

Despite these recommendations, meat consumption in Poland often exceeds dietary guidelines, raising significant public health concerns. While awareness of the health risks associated with excessive meat intake is growing, there is a lack of current, representative research on the actual consumption patterns of red and processed meat in Poland. Previous studies have primarily relied on household budget surveys [25], single 24-h dietary recalls, or frequency-based consumption analyses [3,26,27,28], which do not provide precise quantitative data. This study addresses this gap by presenting comprehensive insights into red and processed meat consumption in Poland, comparing observed intake levels to national dietary recommendations. Importantly, this study also examines the sociodemographic factors influencing adherence to dietary guidelines, including sex, age, place of residence, education level, and financial status. Understanding these factors is essential for tailoring public health recommendations and interventions to specific population groups, thereby improving adherence to dietary guidelines and reducing health risks associated with excessive meat consumption.

The objective of this study was to analyze red and processed meat consumption in Poland and compare the findings to current quantitative Polish food-based dietary guidelines. Additionally, this study aimed to identify sociodemographic factors associated with adherence to these recommendations and to characterize consumer profiles that either meet or exceed recommended intake levels. By addressing these aspects, the study provides evidence to guide public health strategies aimed at reducing excessive meat consumption and promoting healthier dietary habits.

## 2. Materials and Methods

### 2.1. Design

The present study analyzed data from two representative cross-sectional surveys on the dietary habits and nutritional status of adults in Poland, conducted between 2017 and 2020 as part of the National Health Program, with the sample size of 4000 participants, funded by the Ministry of Health. The detailed methodology of these surveys is described in another paper [29].

### 2.2. Sampling

The selection of the respondents was carried out in accordance with the guidelines of the European Food Safety Authority (EFSA) [30], using random sampling from a household address database. To enhance the representativeness and reduce costs, stratified and clustered sampling was employed. At the first stage, 500 statistical areas (clusters) were randomly selected from a total of 34,633 units, with the probability of selection proportional to the population size. Residential buildings within each cluster were then chosen using the TERYT-NOBC register and other sources. Eight buildings were selected per cluster (four for each group), with reserves in case of non-participation. In each selected building, one respondent was randomly chosen.

### 2.3. Sample Size and Representativeness

A sample size of approximately 1067 respondents would be required to achieve a margin of error of no more than 3%, with the proportion of 0.5 and the confidence level of 95%. However, the study in question used a sample size of 4000 individuals, which is significantly larger than the minimum required. This larger sample size reduces the margin of error even further and allows for more detailed subgroup analyses (e.g., by age, sex, education), ensuring that the results are more robust and representative of the population as a whole. A key element of the analytical process involved the application of weights to ensure the sample accurately reflected the distribution of attributes such as the sex, age, place of residence, and education level within the Polish population. The weighting factors were calibrated based on data from the most recent National Population and Housing Census 2021, conducted by Statistics Poland [31], to ensure the representativeness of the sample. The detailed characteristics of the study population are described in our previous paper [32].

### 2.4. Ethical Consideration

The surveys were approved by the Ethics Committee of the Medical University of Warsaw (KBE/163/17 and AKBE/164/17) and were conducted in accordance with the Personal Data Protection Act [33] and the Declaration of Helsinki [34]. Informed consent was obtained from all participants.

### 2.5. Data Collection

The surveys were conducted using computer-assisted personal interviewing (CAPI), with 90% of the interviews being completed using this method. During the COVID-19 pandemic, 10% of the interviews were conducted using computer-assisted telephone interviewing (CATI). The first interview lasted approximately 90 min, while the second interview lasted about 45 min.

### 2.6. Instruments

Two 24-h recalls were used to assess the diet. They were carried out with an interval of at least 5 days. Interviewers, after comprehensive training, conducted interviews according to structured stages, including: indication of products/dishes consumed, assessment of portion sizes and verification of information. The study was conducted on non-consecutive days of the week, including both weekdays and weekends, and at different times of the year to account for variability and seasonality in eating habits. The interviews were carried out using the Dieta 5.0 [35] software recommended for scientific research, using the current Polish database containing information on the content of nutrients in typical products and dishes. Portion sizes were estimated on the basis of an album of photographs of products and dishes [36], as well as on the basis of the size of the packaging of products available on the market. During the interviews, information on physical activity, smoking, alcohol consumption and sociodemographic data were also collected. The information collected was verified and approved on an ongoing basis by the research team nutritionists.

The study focused on the assessment of the consumption of red meat (RM), processed meat (PM) and their combined consumption (Combined Red and Processed Meat—CRPM), including poultry products. White meat is an essential part of the diet. It is also a healthier alternative to red and processed meat. However, the study was deliberately limited to the analysis of the last two groups. This is due to scientific evidence indicating a higher health risk associated with their excessive consumption and the lack of precise recommendations regarding the consumption of white meat.

Daily meat intake in three categories (RM, PM, CRPM) was calculated using the Dieta 5.0 software, which makes it possible to assess the intake of both groups of products as well as ingredients used in dishes and composite products. RM and PM were classified as mutually exclusive categories, as defined by WHO and IARC. CRPM was calculated as the sum of these categories. RM included only unprocessed mammalian muscle meat, such as pork, beef, veal, mutton, and horse meat. PM included meat products that had undergone preservation through salting, smoking, curing, fermentation, or the addition of chemical preservatives: pork cold cuts (including luxury and popular, medium- and fine-ground), poultry cold cuts (popular and luxury, medium- and fine-ground), luxury beef cold cuts, Italian salceson, buckwheat and pate sausage and delicatessen products. The classification was applied as follows: a grilled pork chop was classified as RM since it underwent only thermal processing, whereas pork cold cuts, for example, were classified as PM due to the use of curing, smoking, or preservatives.

Additionally, the share of PM in CRPM was calculated. CRPM intake was evaluated based on dietary guidelines recommending 350–500 g of red and processed meat per week, corresponding to 50–70 g/day. Intake was categorized into three levels: below 50 g/day, 50–70 g/day, and above 70 g/day [1,22,24].

### 2.7. Statistical Analysis

All statistical calculations were conducted within the framework of a weighted analysis to ensure the findings were representative of a broader population structure. This adjustment accounted for potential biases related to demographic distribution and enhanced the generalizability of the results. In the statistical evaluation of the study, inferential analyses were conducted using null hypothesis testing. Descriptive statistics, including means, 95% confidence intervals (CIs), and specific percentiles (5th, 10th, 25th, 50th, 75th, 90th, and 95th), were employed to characterize the distribution of daily consumption levels of RM, PM, and CRPM among study participants. This approach provides a detailed and nuanced depiction of the variability in meat consumption, and it is important to note that these percentiles reflect the distribution of consumption values rather than segmenting the study population itself.

A comparative analysis of CRPM between sexes was conducted using the Student’s *t*-test. Chi-squared tests for independence were used to assess the proportion of individuals exceeding recommended meat intake levels.

A detailed examination of CRPM consumption across different age and sex groups was conducted using Analysis of Variance (ANOVA). A two-factor ANOVA was used to assess the effects of sex and age group, as well as the interaction between those factors, on CRPM. Post-hoc analyses, such as the Games–Howell test, revealed specific differences between age groups, particularly between the youngest and oldest cohorts.

The statistical analysis employed logistic regression to examine the correlation of selected sociodemographic factors, such as sex, age, education, place of residence, and financial status, on adherence to the dietary recommendation of consuming ≤70 g of CRPM per day. Odds ratios (ORs) and 95% CIs were calculated to estimate the likelihood of adherence for each predictor. The model’s parameters were estimated using the maximum likelihood method. The overall fit of the model was assessed using McFadden’s R^2^.

All statistical analyses were executed using STATISTICA™ version 13.3 (TIBCO^®^ Software Inc., Palo Alto, CA, USA), with a predetermined alpha level of 0.05 for the rejection of the null hypothesis in all statistical tests.

## 3. Results

### 3.1. Average Meat Consumption in the General Polish Population

The analysis of data on meat intake in the general population revealed that the average daily intake of RM was 64.0 g (95% CI: 60.7–67.2 g). In case of PM, it was 75.0 g (95% CI: 71.9–78.2 g). Total CRPM intake averaged at 134.0 g per day (95% CI: 134.2–143.8 g). The median intake of RM was 48.6 g/day, PM 53.6 g/day, and CRPM 116.4 g/day.

In addition, on average, PM constituted 59.37% of the CRPM intake (95% CI: 57.89–60.85%). In half of the studied population, PM accounted for over 50% of the CRPM intake (53.6 g/day). For 25% of the population, PM constituted as much as 100% of total meat consumption, which means that unprocessed red meat was not consumed in the group (Table 1).

### 3.2. Sex and Age-Related Differences in Average Meat Consumption

The mean RM intake in men was significantly higher than in women (74.4 ± 81.7 g/day vs. 47.3 ± 55.3 g/day; t = −11.6; *p* < 0.001). Similarly, PM intake was higher in the group of men compared to women (96.5 ± 78.5 g/day vs. 58.4 ± 62.8 g/day; t = −16.1; *p* < 0.001). A significant sex-related difference was also shown for CRPM consumption with higher intake noted in men (171 ± 120 g/day vs. 106 ± 86.6 g/day; t = 18.5; *p* < 0.001). Conversely, the proportion of PM to CRPM did not differ significantly between the sexes (61.4% ± 31.0% in men vs. 60.9% ± 35.9% in women; t = −0.464; *p* = 0.643).

RM intake in men reached the highest values in the 19–34 age group (91.0 g/day) and remained high in all subsequent age groups. On average, women consumed less RM, with the mean values ranging from 43.6 g/day in the 75+ age group to 52.8 g/day in the 19–34 age group. Similarly, PM intake was higher in men, especially in the 35–44 (104.0 g/day) and 45–54 (107.3 g/day) age groups. On average, women consumed less PM, with the mean intake ranging from 46.4 g/day in the 75+ age group to 61.8 g/day in the 45–54 age group. CRPM intake was also significantly higher in men, reaching the highest values in the 45–54 age group (196.7 g/day). In women, the lowest CRPM intake values were recorded in the 75+ group (89.9 g/day), and the highest in the 45–54 age group (109.6 g/day). As regards the share of PM in CRPM, sex-related differences were less pronounced, with the values similar across age groups. The mean values of PM/CRPM intake for men ranged from 53.71% in the 19–34 age group to 62.26% in the 55–64 group. The respective values for women ranged from 57.53% in the 75+ group to 62.17% in the 45–54 age group (Table 2). Detailed consumption percentiles for red meat, processed meat, combined red and processed meat, and the proportion of processed meat in total meat intake are provided in the Supplementary Materials (Tables S1–S4).

### 3.3. Age-Related Variation in Meat Consumption

A one-way analysis of variance (ANOVA) confirmed a significant difference in RM intake in different age groups (F(5.1345) = 8.81, *p* < 0.001), with generally higher RM intake in younger groups. The Games–Howell post-hoc test revealed that significant differences occurred mainly between the youngest and oldest age groups. Similar results were obtained for PM (F(5.1387) = 5.37, *p* < 0.001) showing that middle-aged study participants consumed more PM compared to other age groups. Significant differences mainly occurred between middle-aged individuals (35–54 years) and other age groups. In the case of CRPM consumption, the age group was also a significant factor differentiating intake (F(5.1363) = 9.81, *p* < 0.001), with middle-aged respondents consuming more meat in general compared to younger and older groups. The most marked differences were found between the 45–54 age group and other groups. The PM/CRPM ratio was also characterized by significant age-related differences (F(5.1358) = 4.05, *p* = 0.001). Older age groups (65–74.9 and 75+) had a higher proportion of PM in the diet than younger adults (19–34 years). The most marked differences were observed between the youngest and oldest age groups.

### 3.4. Sex and Age Interactions in the Consumption of Red and Processed Meat

A two-way ANOVA was conducted to examine the effects of sex and age group on the CRPM, as well as the interaction between those factors. The analysis revealed significant main effects of both sex and age on meat consumption. On average, men consumed significantly more CRPM than women across all age groups. Additionally, younger age groups were found to consume higher amounts of meat compared to older age groups.

**Figure 1:**
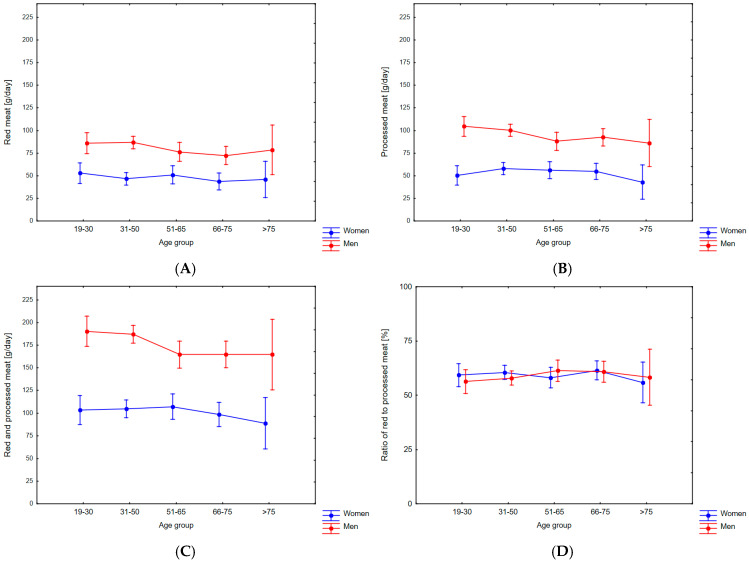
Sex and age-related differences in red and processed meat consumption: mean values with 95% confidence intervals ((**A**) red meat, (**B**) processed meat, (**C**) combined red and processed meat, (**D**) ratio of red to processed meat).

However, the interaction effect between sex and age group was not statistically significant for either RM (*p* = 0.367) (Figure 1A) or PM (*p* = 0.393) (Figure 1B). This indicates that, while there are clear differences in consumption based on sex and age independently, the pattern of meat consumption across age groups did not differ significantly between men and women.

Similarly, the interaction effect for combined red and processed meat consumption (CRPM) was not significant (*p* = 0.119) (Figure 1C), and no significant interaction was found for the proportion of processed meat in total meat consumption (*p* = 0.618) (Figure 1D). These results suggest that the sex differences in meat consumption remain consistent across age groups.

### 3.5. Adherence to Meat Consumption Guidelines by Sex and Age

The analysis of the implementation of the CRPM intake recommendations showed that the majority of the population (69.43%) consumed over 70 g of meat per day, which exceeds the recommended limit. Only 21.88% of the respondents consumed less than 50 g of meat per day, and in 8.69%, the consumption ranged from 50 to 70 g/day.

The analysis of CRPM intake across age groups showed that the 45–54 age group included the highest percentage of individuals (74.03%) exceeding the recommended meat intake (>70 g/day). The percentage was the lowest and amounted to 60.40% in the oldest age group (75+). As regards younger respondents (19–34 years), 22.52% of them consumed below 50 g of meat per day, which was the lowest percentage of adherence to meat consumption recommendations compared to other age groups (Table 3).

The analysis of the results revealed significant differences between women and men in the implementation of recommendations regarding meat consumption (χ^2^ = 132.526; *p* < 0.001). A higher percentage of men (81.55%) exceeded the recommended intake of 70 g of meat per day compared to women (58.29%). The consumption of less than 50 g of meat per day was significantly more common in women (30.65%) compared to men (12.33%). A detailed analysis of the implementation of the recommendations regarding CRPM intake demonstrated differences depending on sex and age group. It was observed that men were much more likely to exceed the recommended level of 70 g of meat per day, while women were more likely to consume below 50 g/day. The highest percentage of individuals exceeding the recommendations was noted in the 19–34 age group, and the lowest in the 75+ group (Table 4).

### 3.6. Sociodemographic Determinants of Adherence to Red and Processed Meat Intake Recommendations

Logistic regression analysis revealed that the model was statistically significant, although it explained a relatively small part of the variance (R^2^McF = 0.0496, χ^2^ = 229, df = 9, *p* < 0.001). The analysis indicated that sex, age, and tertiary education were significant predictors of adherence to the meat intake recommendation of 70 g daily or less. Men were less likely to adhere to the recommendation compared to women (OR = 0.36, 95% CI: 0.31, 0.42), while older people and those with tertiary education were more likely to adhere (OR = 1.01, 95% CI: 1.00, 1.01; OR = 1.38, 95% CI: 1.08, 1.76). The place of residence and financial situation had no significant impact on adherence to dietary recommendations (Table 5).

## 4. Discussion

The study results indicate a significant exceedance of the recommended reference values of CRPM consumption in the Polish population, which increases health risks, especially in the context of PM which dominated in the diets of the respondents. The analysis of sociodemographic factors that influence consumption patterns was crucial: men consumed more meat than women. Higher consumption was also noted in younger people and those with lower levels of education. In light of the consensus positions of international organizations recommending CRPM limitation [1,9,24], such a high CRPM intake poses a serious threat to public health. The study provides up-to-date information on meat consumption in Poland, which is crucial for the development of dietary guidelines and public health interventions. The study also may underline more individualized recommendations, taking account of differences in meat consumption between sociodemographic groups.

The results of the study indicate that the average daily intake of CRPM was 139.0 g in the general population, including RM 64.5 g and PM 75.0 g. The results emphasize a favorable decline in meat consumption in Poland over the past 25 years. Szponar et al. [26] estimated the average consumption of red meat, poultry, offal and their products at 245 g/day based on a one-time dietary recall conducted in a representative group of adult Polish residents. During the study period, men consumed 332 g of meat and its products per day, and women consumed 175 g/day. The WOBASZ 2003–2005 (Multicenter Population Health Research) study [27] conducted several years later was based on the same methodology for assessing food intake. The mean total meat intake (including white meat) was estimated at 260 g/day in men and 142 g/day in women, with a significant decrease in consumption, especially in men. Comparing the results of our study to current representative European studies, in which the average meat consumption among adults ranged from 75 g/day to 233 g/day, it can be seen that Poland, with the result of 139.0 g/day, ranked in the middle of the range. Meat consumption in Poland was higher than in countries such as Sweden (75–93 g/day), the Netherlands (110 g/day) or Ireland (117–134 g/day), but lower than in countries such as Germany (85–233 g/day), Finland (191–211 g/day) or the Czech Republic (187 g/day) [37]. In the United States, it is estimated that daily meat intake in people aged 2 years and older ranged from 125.9 to 166.5 g, which makes it difficult to compare with the present results, which only included the adult population [38]. Discrepancies in meat consumption between Poland and other countries may result from cultural differences, dietary traditions, product availability and health awareness. In countries with stronger health and environmental trends, such as Sweden or Germany, meat consumption is lower, while in Poland culinary traditions and relatively lower meat prices favor higher consumption. Changes in eating habits are gradual in Poland, although the awareness of health risks related to excessive meat consumption is growing. It is difficult to compare the results of our study to other studies conducted in Poland which were based on the analysis of household budgets due to significant methodological differences. Nevertheless, positive changes in the structure and amount of meat consumption in Poland over the past two decades have been confirmed. The research of Statistics Poland showed that, in 2021, the total consumption of meat per person in a household was 229.4 g/day, including the consumption of poultry at 48.3 g. It had decreased by 27.9 g per day since 2000. Moreover, the consumption of cold cuts had decreased from 75 g to 63.7 g/day over the period [39]. The trend of reducing meat consumption observed in Poland is also visible in the United Kingdom, where the average daily meat consumption per person decreased from 103.7 g to 86.3 g in the years 2008–2019. The reduction included both red meat consumption, which decreased by 13.7 g (from 37.4 g/d to 23.7 g/d), and processed meat consumption, which decreased by 7.0 g (from 33.8 g/d to 26.8 g/d) [40]. In case of the American population, the analysis of the trend of processed meat consumption in American adults over the past 18 years showed that it remained virtually unchanged at 182 g/week in 1999–2000 and 187 g/week in 2015–2016 [41]. The present study showed the average intake of RM was 64.0 g/day. As regards processed meat, it was 75.0 g/day. The analysis presenting the results of representative studies conducted in the United States, Canada and Mexico in 2013–2016 showed that the median consumption of red and processed meat was at the following levels: in the USA—79.4 g/day, in Canada—79.0 g/day, and in Mexico—62.5 g/day. The median consumption of processed meat was: USA—44.5 g/day, Canada—41.8 g/day, Mexico—40.0 g/day [42]. Our study showed that processed meat accounted for 59.37% of total meat consumption in Poland, which considerably exceeded the international dietary guidelines that recommend minimizing its consumption due to its association with chronic diseases such as heart disease, tumors or type 2 diabetes. It is particularly alarming that for 25% of the population PM accounted for 100% of total meat consumption. Such a level of consumption is associated with significant costs for the healthcare system due to the treatment of diet-related diseases. Our observation was confirmed by the research of Stoś et al. conducted on the basis of the analysis of data from household budget surveys in 2000, 2010 and 2020 [28]. Notably, the pattern of meat consumption has changed significantly over the past quarter of a century. Based on the results of the study by Szponar et al., at the end of the 20th century, meat products accounted for 34.9% of total meat consumption [26]. It may have resulted from the smaller assortment of meat products on the market, as well as from habits related to the long-term rationing of meat in Poland and the preparation of most dishes at home. Rationing meat and its products was in force in Poland in 1981–1989 [43]. Currently, the situation has been reversed, and meat products dominate the diet of Polish people. This situation requires educational activities aimed not only at limiting the consumption of meat and its products but also promoting unprocessed high-quality meat and trends supporting plant-based diets. Increasing the awareness of the benefits of consuming more plant-based foods, including plant-based proteins, may support health and contribute to global trends in carbon footprint reduction and sustainable agriculture, promoting healthier and greener eating habits at the individual and societal levels.

This study indicates that, among the analyzed sociodemographic features, age, gender, and education level had a significant impact on meat consumption patterns in Poland, while place of residence and financial status did not show a statistically significant association. Sex is an important factor playing a key role in consumption differences. Men consumed significantly more meat than women, which was confirmed in our study, where the average daily meat intake was 171 g in men compared to 106 g in women. Similar observations were made in the previously mentioned WOBASZ II study [27]. At the end of the 20th century, men in Poland consumed an average of 332 g of meat and its products per day, and women consumed 175 g/day, which indicates the existence of a favorable trend. Furthermore, research on the frequency of meat consumption indicated clear differences between the sexes, reporting a higher frequency of meat consumption in men [28,44,45]. The results obtained over the years have clearly confirmed the significant influence of sex on meat consumption patterns in Polish society. Similar patterns may be observed in other European countries, where men also tend to eat more meat. A review of data from ten European countries revealed that the average total meat consumption was 84–218 g/day in men, and 64–163 g/day in women [37]. The German National Nutrition Survey II also showed significantly higher meat consumption in men (155 g/day) compared to women (87 g/day). Similar trends were also observed outside Europe. In the United States and Canada, the consumption of unprocessed red meat was more common in men, with a respective difference of 7.8% and 8.6% compared to women [42]. Our study demonstrated that, compared to women, men were less likely to follow the recommendation to consume 70 g of meat or less per day. This may be due to the fact that higher meat consumption in men can be attributed to both greater energy requirements and traditional cultural norms that equate meat, especially red meat, with masculinity. This kind of perception has its roots in historical and cultural messages, where meat symbolizes strength, vitality and wealth. The consumption of meat by men is often treated as an expression of masculinity, which affects their food preferences and leads to higher meat consumption compared to women [46]. Conversely, women more commonly choose plant-based diets, guided by both health and ethical considerations. Such differences in dietary choices may result from different attitudes towards health—women are more likely to pay attention to the health aspects of their diet, while men consume more meat, treating it as a confirmation of their masculinity [47,48]. Social norms and cultural pressure further reinforce the differences, encouraging men to consume more meat, while women are more likely to choose healthier and more sustainable alternatives.

Age is another important variable that plays a key role in shaping meat consumption patterns. The highest meat consumption, of both RM and PM, was observed in younger age groups, especially in men, where it peaked at the age of 19–34. In men, meat consumption remained at a relatively high level for most of their lives, which indicated stable eating habits in this group. In contrast, in women, meat consumption was lower in all age groups and tended to decrease with age, with the highest values recorded in the 45–54 age group. Such differences may reflect changing nutritional needs, lifestyle, and increasing health awareness at an older age. The analysis of the present results in relation to research by Szponar et al. is difficult due to different age ranges of the study populations. Notably, the youngest women, i.e., at the age of 19–25, consumed the least meat and its products (153 g/day), this amount increased to 182 g/day at the age of 26–60 and slightly decreased (170 g/day) after the age of 60. In the group of men, age-related changes were more pronounced. Moreover, the youngest men consumed the most meat and meat products per day (366 g/day). The value decreased slightly until the age of 60 (345 g/day) and dropped significantly in the group of the oldest men (255 g/day). Such changes may have resulted from a change in lifestyle in the senior period and lower energy demand after retirement (after 65 years of age) [26]. The influence of age on meat consumption was also observed in most European countries. Adults aged 18–69 years consumed more meat (from 93 to 233 g/day) than older people (over 65 years of age), whose intake ranged from 75 to 191 g/day [37]. In Germany, meat consumption tended to decrease with age [49]. Studies in the UK revealed that older people consumed less energy from meat than younger adults [40]. In Mexico and Canada, older generations consumed less meat, but in North America the differences were not statistically significant [42].

Our study showed that sex, age and the level of education were important predictors of adherence to the meat consumption recommendations. Our results fit into the broader context of research, which indicates the key role of education in shaping eating habits. The impact of education on the frequency of meat consumption was noted in the study by Stoś et al., where a higher level of education was associated with a lower frequency of sausage and bacon consumption, and, in the case of women, also pork. However, in men, the frequency of consumption of beef, veal and mutton, which are considered more expensive types of meat in Poland, increased with an increase in the level of education [28]. Studies by other authors also indicated that people with higher education consumed significantly less meat compared to people with a lower level of education, which is especially visible in case of processed meat [42,50,51]. It may be due to higher awareness of the health benefits of reducing meat consumption and greater environmental awareness in these social groups. Additionally, such people have better access to healthy foods, which promotes healthier choices [52,53]. As regards high-income countries, such as the United States and Canada, education was confirmed to be a stronger indicator of diet quality than income [42]. Education-related differences in meat consumption may perpetuate health and nutrition inequalities, which highlights the need to implement preventive policies that take account of these disparities.

### 4.1. Practical Implications

The results of this study indicate that the consumption of red and processed meat markedly exceeds both national and international health recommendations. Such a level of consumption increases the risk of chronic diseases, which creates challenges for the healthcare system. Therefore, public health policy should focus on the implementation of strategies to reduce the consumption of processed meat in particular. These findings highlight the importance of developing targeted, evidence-based public health strategies to combat the excessive consumption of meat, particularly processed meat, which poses significant health risks. Public health campaigns should focus on promoting awareness of the risks associated with high meat intake and encourage dietary shifts toward plant-based foods and healthier protein sources. Culturally adapted interventions are crucial, particularly for men and younger populations, to address the social factors that drive high meat consumption. Educational programs, community outreach, and media campaigns might play a pivotal role in reshaping dietary preferences and reducing the appeal of meat-heavy diets.

Effective education campaigns may include promoting healthier alternatives, such as plant-based protein sources, and highlighting the importance of selecting unprocessed meats. Policy initiatives such as food labeling and the introduction of taxes on processed meat may also be helpful in limiting consumption. Due to the clear differences in meat consumption across different social groups, it is necessary to implement personalized interventions, especially targeted at men and younger populations, which are characterized by higher meat consumption. Further research is crucial for monitoring the effectiveness of implemented strategies and changes in meat consumption patterns in Poland.

### 4.2. Strengths and Limitations

The strengths of the study include several key elements that significantly increase its reliability and make it possible to generalize the results to the entire population of Poland. Firstly, the study was conducted on a large, representative sample, which allows for a detailed analysis of meat consumption in various sociodemographic groups. The use of weighted analysis, taking account of the demographic structure of the studied population, additionally increases the precision of the results and makes it possible to refer them to the general public. As a result, the data obtained are not only accurate, but also accurately reflect real consumption habits in the context of health recommendations. The value of the study also lies in the use of a repeated 24-h dietary recall, which is the recommended method in food intake assessment studies.

The limitations of the study include, first of all, the dependence on data based on the memory of the respondents, which may lead to the underestimation of meat consumption, especially as regards products perceived as less healthy, i.e., processed meat. Moreover, the study did not take account of all factors that might affect meat consumption patterns, such as culinary habits or individual dietary preferences. Including such aspects could provide a more complete picture of meat consumption and its relationship with health.

## 5. Conclusions

This study offers critical insights into the patterns of red and processed meat consumption in Poland, revealing significant sociodemographic differences that have important public health implications. With two-thirds of the population exceeding the recommended daily limit of 70 g of meat, the findings signal an urgent need to address dietary behaviors that contribute to excessive meat consumption.

The analysis identified that the profile of individuals most likely to adhere to meat consumption recommendations includes women, older individuals, and those with higher education. In contrast, men, younger individuals, and those with lower educational attainment were less likely to meet the recommended intake limits. These results underscore the need for tailored public health interventions aimed at high-risk groups, particularly men and younger populations, who consume significantly more meat. Younger and middle-aged men exhibited the highest levels of meat consumption, reflecting cultural norms that associate meat, especially red meat, with masculinity. Addressing these cultural associations is crucial for promoting healthier, more balanced diets.

Policy measures, such as clearer food labeling, taxation on processed meat products, and subsidies for healthier food options may also be effective tools in encouraging dietary changes. Such strategies should be supported by further research to assess their long-term effectiveness in reducing meat consumption and improving public health outcomes. By integrating these findings into public health planning, policymakers may better address the health challenges associated with excessive meat consumption and foster a healthier, more sustainable dietary environment in Poland.

## Figures and Tables

**Table 1:** Consumption of individual categories of meat in the total population.

Type of Meat	Mean	−95% CI	+95% CI	Percentiles						
				5th	10th	25th	50th	75th	90th	95th
RM [g/day]	64.0	60.7	67.2	0.0	0.0	0.0	48.6	93.7	153.0	203.5
PM [g/day]	75.0	71.9	78.2	0.0	5.7	21.9	53.6	109.1	175.7	214.4
CRPM [g/day]	139.0	134.2	143.8	6.3	17.1	55.8	116.4	192.7	282.7	347.6
PM/CRPM [%]	59.4	57.9	60.9	0.0	11.6	31.1	57.9	100.0	100.0	100.0

RM—red meat, PM—processed meat, CRPM—combined red and processed meat, PM/CRPM—ratio of processed meat and the total consumption of red and processed meat.

**Table 2:** Mean of red meat, processed meat, combined red and processed meat consumption (g/day), and proportion of processed meat in total meat intake (%) by sex and age.

Sex/Age Group (Years)	N	Mean ± SD			
		RM	PM	CRPM	PM/CRPM
Women:					
19.0–34.9	240	52.8 ± 67.2	56.0 ± 66.6	108.8 ± 99.1	58.7 ± 36.5
35.0–44.9	204	46.4 ± 51.5	54.1 ± 59.5	100.5 ± 82.0	59.3 ± 36.5
45.0–54.9	198	47.8 ± 49.0	61.8 ± 63.6	109.6 ± 85.7	62.2 ± 33.2
55.0–64.9	129	49.5 ± 63.5	52.1 ± 53.6	101.5 ± 88.8	58.4 ± 37.9
65.0–74.9	231	43.9 ± 51.3	53.4 ± 56.4	97.3 ± 76.6	61.0 ± 36.9
75+	65	43.6 ± 58.0	46.4 ± 56.3	90.0 ± 79.8	57.5 ± 40.8
Men:					
19.0–34.9	244	91.0 ± 92.9	95.8 ± 81.2	186.8 ± 128.8	53.7 ± 32.7
35.0–44.9	229	80.9 ± 76.6	104.0 ± 78.9	184.9 ± 113.7	59.8 ± 30.7
45.0–54.9	164	89.4 ± 96.0	107.3 ± 79.8	196.7 ± 128.5	59.9 ± 29.9
55.0–64.9	107	65.4 ± 75.2	77.6 ± 58.9	143.1 ± 100.0	62.3 ± 32.2
65.0–74.9	201	75.2 ± 87.1	93.1 ± 77.8	168.3 ± 117.4	61.1 ± 31.3
75+	36	69.8 ± 74.6	85.2 ± 67.5	155.0 ± 86.6	60.4 ± 36.0
Total:					
19.0–34.9	484	72.0 ± 83.3	76.1 ± 76.9	148.1 ± 121.4	56.2 ± 34.7
35.0–44.9	433	64.7 ± 68.1	80.5 ± 74.6	145.1 ± 108.4	59.6 ± 33.5
45.0–54.9	362	66.7 ± 77.1	82.4 ± 74.8	149.1 ± 115.6	61.2 ± 31.7
55.0–64.9	236	56.7 ± 69.3	63.7 ± 57.4	120.4 ± 96.1	60.2 ± 35.4
65.0–74.9	432	58.4 ± 71.9	71.9 ± 70.0	130.3 ± 103.8	61.0 ± 34.4
75+	101	52.9 ± 65.2	60.2 ± 63.0	113.2 ± 87.6	58.5 ± 39.0

RM—red meat, PM—processed meat, CRPM—combined red and processed meat, PM/CRPM—proportion of processed meat in total meat intake; SD—standard deviation.

**Table 3:** Implementation of recommendations for combined red and processed meat consumption depending on age in the general population.

Age Group (Years)	<50 g/Day		50–70 g/Day		>70 g/Day		χ^2^	*p*-Value *
	N	%	N	%	N	%		
19.0–34.9	109	22.52	40	8.26	335	69.21	25.554	0.004
35.0–44.9	95	21.94	31	7.16	307	70.90		
45.0–54.9	51	14.09	43	11.88	268	74.03		
55.0–64.9	62	26.27	21	8.90	153	64.83		
65.0–74.9	103	23.84	31	7.18	298	68.98		
75+	28	27.72	12	11.88	61	60.40		

* Pearson’s chi-squared test.

**Table 4:** Implementation of recommendations for combined red and processed meat consumption depending on the sex and age.

Implementation of Recommendations	Age Group (Years)	Women		Men		Total	
		N	%	N	%	N	%
<50 g/day	19.0–34.9	76	23.24	33	27.27	109	24.33
	35.0–44.9	64	19.57	31	25.62	95	21.21
	45.0–54.9	41	12.54	10	8.26	51	11.38
	55.0–64.9	47	14.37	15	12.40	62	13.84
	65.0–74.9	76	23.24	27	22.31	103	22.99
	75+	23	7.03	5	4.13	28	6.25
	Total	327	30.65	121	12.33	448	21.88
50–70 g/day	19.0–34.9	23	19.49	17	28.33	40	22.47
	35.0–44.9	20	16.95	11	18.33	31	17.42
	45.0–54.9	37	31.36	6	10.00	43	24.16
	55.0–64.9	12	10.17	9	15.00	21	11.80
	65.0–74.9	18	15.25	13	21.67	31	17.42
	75+	8	6.78	4	6.67	12	6.74
	Total	118	11.06	60	6.12	178	8.69
>70 g/day	19.0–34.9	141	22.67	194	24.25	335	23.56
	35.0–44.9	120	19.29	187	23.38	307	21.59
	45.0–54.9	120	19.29	148	18.50	268	18.85
	55.0–64.9	70	11.25	83	10.38	153	10.76
	65.0–74.9	137	22.03	161	20.13	298	20.96
	75+	34	5.47	27	3.38	61	4.29
	Total	622	58.29	800	81.55	1422	69.43

**Table 5:** Logistic regression analysis of the impact of selected sociodemographic factors on adherence to the recommendations on combined red and processed meat consumption at the level of ≤70 g/day.

			95% Confidence Interval			
Predictor	Estimate	Odds Ratio	Lower	Upper	Z-Score	*p*-Value
Intercept	−0.65	0.52	0.35	0.77	−3.268	0.001
Sex						
Man vs. Women	−1.02	0.36	0.31	0.42	−13.108	<0.001
Age	0.01	1.01	1.00	1.01	3.076	0.002
Place of residence:						
town <20 thousand vs. village	−0.01	0.99	0.79	1.24	−0.075	0.940
town 20–100 thousand vs. village	−0.11	0.90	0.73	1.10	−1.043	0.297
city >100 thousand vs. village	0.09	1.10	0.92	1.31	1.005	0.315
Education:						
Secondary vs. primary/junior high/vocational	0.04	1.04	0.88	1.22	0.413	0.680
Tertiary vs. primary/junior high/vocational	0.32	1.38	1.08	1.76	2.582	0.010
Financial situation						
Average vs. poor	−0.22	0.80	0.63	1.02	−1.825	0.068
Good vs. poor	−0.02	0.98	0.73	1.31	−0.133	0.894

## Data Availability

The original contributions presented in the study are included in the article/Supplementary Material, further inquiries can be directed to the corresponding authors.

## Supplementary Materials

The following supporting information can be downloaded at: https://www.mdpi.com/article/10.3390/nu17050790/s1, Table S1. Mean and percentiles of red meat consumption (g/day) by sex and age. Table S2. Mean and percentiles of processed meat consumption (g/day) by sex and age. Table S3. Mean and percentiles of combined red and processed meat consumption (g/day) by sex and age. Table S4. Mean and percentiles of the share of processed meat in combined red and processed meat consumption (%) by sex and age.

## Abbreviations

RM	red meat
PM	processed meat
CRPM	combined red and processed meat
PM/CRPM	ratio of processed meat and the total consumption of red and processed meat
