# Supplementary material for: Adherence to Dietary Recommendations for Red and Processed Meat in Poland: Insights from the 2017–2020 National Nutrition Survey
Source: Nutrients. 2025 Feb 25;17(5):790. doi: 10.3390/nu17050790 (PMC11901433; doi:10.3390/nu17050790)
Supplement: Supplementary file 1 [file nutrients-17-00790-s001.zip › nutrients-3481164-supplementary.pdf]

Table S1. Mean and percentiles of red meat consumption (g/day) by sex and age

Sex / Age group (years)	N	Mean	-95% CI	+95% CI	Percentiles						
					5th	10th	25th	50th	75th	90th	95th
Women:											
19.0-34.9	240	52.8	44.2	61.3	0.0	0.0	0.0	40.0	76.5	132.7	231.0
35.0-44.9	204	46.4	39.3	53.5	0.0	0.0	0.0	37.2	79.3	110.7	141.5
45.0-54.9	198	47.8	40.8	54.8	0.0	0.0	0.0	42.3	76.5	118.4	146.4
55.0-64.9	129	49.5	38.4	60.5	0.0	0.0	0.0	24.6	81.8	137.2	174.9
65.0-74.9	231	43.9	37.2	50.5	0.0	0.0	0.0	37.7	76.5	104.6	127.8
75+	65	43.6	29.2	57.9	0.0	0.0	0.0	20.5	69.7	127.8	147.9
Men:											
19.0-34.9	244	91.0	79.3	102.7	0.0	0.0	17.6	71.6	139.1	196.7	281.3
35.0-44.9	229	80.9	70.9	90.9	0.0	0.0	0.0	76.5	120.4	175.0	238.7
45.0-54.9	164	89.4	74.7	104.2	0.0	0.0	15.5	71.8	125.6	194.1	246.5
55.0-64.9	107	65.4	51.0	79.8	0.0	0.0	0.0	53.3	90.6	168.3	217.4
65.0-74.9	201	75.2	63.1	87.3	0.0	0.0	0.0	57.4	108.0	163.7	223.7
75+	36	69.8	44.6	95.0	0.0	0.0	0.0	42.5	124.6	159.9	243.5
Total:											
19.0-34.9	484	72.0	64.6	79.5	0.0	0.0	0.0	50.8	107.7	173.9	248.8
35.0-44.9	433	64.7	58.2	71.1	0.0	0.0	0.0	57.4	102.0	146.2	186.4
45.0-54.9	362	66.7	58.7	74.6	0.0	0.0	0.0	49.3	94.3	155.3	190.4
55.0-64.9	236	56.7	47.8	65.6	0.0	0.0	0.0	40.0	82.6	151.9	197.2
65.0-74.9	432	58.4	51.6	65.2	0.0	0.0	0.0	48.4	82.2	141.5	185.9
75+	101	52.9	40.1	65.8	0.0	0.0	0.0	30.8	85.0	145.7	159.9

CI – confidence interval

Table S2. Mean and percentiles of processed meat consumption (g/day) by sex and age

Sex / Age group (years)	N	Mean	-95% CI	+95% CI	Percentiles						
					5th	10th	25th	50th	75th	90th	95th
Women:											
19.0-34.9	240	56.0	47.6	64.5	0.0	1.3	12.5	35.9	70.6	138.9	194.3
35.0-44.9	204	54.1	45.9	62.3	0.0	1.9	12.1	34.7	69.3	138.0	182.0
45.0-54.9	198	61.8	52.9	70.7	3.8	8.5	20.5	37.5	81.5	138.2	212.3
55.0-64.9	129	52.1	42.8	61.4	0.0	1.3	14.4	36.5	70.0	147.2	161.4
65.0-74.9	231	53.4	46.1	60.8	0.0	3.2	14.4	34.1	72.5	121.4	176.4
75+	65	46.4	32.5	60.3	0.0	0.0	3.8	30.0	53.8	154.4	183.1
Men:											
19.0-34.9	244	95.8	85.6	106.1	0.0	8.9	34.5	79.6	143.8	202.6	232.9
35.0-44.9	229	104.0	93.7	114.2	5.7	16.7	47.8	96.2	147.7	191.1	220.9
45.0-54.9	164	107.3	95.0	119.6	10.8	20.7	53.5	95.1	139.7	223.6	256.7
55.0-64.9	107	77.6	66.4	88.9	3.8	10.2	38.7	69.8	102.5	154.3	196.5
65.0-74.9	201	93.1	82.3	104.0	3.1	19.2	38.3	81.1	124.1	176.4	219.3
75+	36	85.2	62.4	108.1	0.0	2.5	19.2	80.0	132.8	182.3	195.1
Total:											
19.0-34.9	484	76.1	69.2	82.9	0.0	1.3	17.5	51.0	110.2	187.8	223.0
35.0-44.9	433	80.5	73.4	87.5	1.1	7.9	23.4	58.8	118.7	182.0	215.1
45.0-54.9	362	82.4	74.7	90.1	5.7	11.3	26.3	59.6	113.5	182.7	234.9
55.0-64.9	236	63.7	56.3	71.0	0.0	4.3	21.3	49.3	87.0	147.2	177.5

65.0-74.9	432	71.9	65.3	78.5	0.0	7.6	22.1	50.9	107.1	166.7	206.1
75+	101	60.2	47.8	72.7	0.0	0.0	7.5	39.3	93.5	171.2	184.9

CI – confidence interval

Table S3. Mean and percentiles of combined red and processed meat consumption (g/day) by sex and age

Sex / Age group (years)	N	Mean	-95% CI	+95% CI	Percentiles						
					5th	10th	25th	50th	75th	90th	95th
Women:											
19.0-34.9	240	108.8	96.2	121.4	0.0	9.5	32.3	90.9	149.1	236.6	289.3
35.0-44.9	204	100.5	89.2	111.8	1.1	11.3	35.1	83.4	146.8	198.5	256.4
45.0-54.9	198	109.6	97.6	121.6	8.5	13.3	54.2	90.9	146.9	220.8	289.0
55.0-64.9	129	101.5	86.1	117.0	0.0	6.9	33.8	84.4	160.7	243.0	264.2
65.0-74.9	231	97.3	87.4	107.2	7.5	12.5	35.2	91.2	134.6	195.5	234.9
75+	65	90.0	70.2	109.8	0.0	1.7	25.5	75.5	133.1	180.4	234.2
Men:											
19.0-34.9	244	186.8	170.5	203.0	23.1	39.1	90.4	169.3	253.3	352.4	441.6
35.0-44.9	229	184.9	170.1	199.7	18.8	35.6	114.4	171.3	249.0	327.4	361.0
45.0-54.9	164	196.7	176.9	216.5	40.0	79.0	114.1	162.9	256.0	358.3	421.3
55.0-64.9	107	143.1	123.9	162.2	21.3	38.7	74.6	114.5	199.5	287.0	325.9
65.0-74.9	201	168.3	152.0	184.6	22.7	44.0	85.1	148.1	233.5	324.6	394.2
75+	36	155.0	125.7	184.3	13.1	25.5	80.2	158.3	236.0	260.1	279.8
Total:											
19.0-34.9	484	148.1	137.3	159.0	3.4	13.6	53.3	125.1	209.0	307.8	401.9
35.0-44.9	433	145.1	134.9	155.4	2.8	21.6	58.0	135.0	200.0	291.2	333.9
45.0-54.9	362	149.1	137.1	161.0	10.9	29.6	67.1	120.4	207.2	289.0	365.2
55.0-64.9	236	120.4	108.0	132.7	1.9	13.5	48.5	98.9	175.7	258.2	302.7
65.0-74.9	432	130.3	120.5	140.2	10.0	17.3	52.0	107.9	178.2	265.9	334.0
75+	101	113.2	95.9	130.5	1.3	3.8	41.1	102.0	172.4	239.1	274.6

CI – confidence interval

Table S4. Mean and percentiles of the share of processed meat in combined red and processed meat consumption (%) by sex and age

Sex / Age group (years)	N	Mean	-95% CI	+95% CI	Percentiles						
					5th	10th	25th	50th	75th	90th	95th
Women:											
19.0-34.9	240	58.7	54.0	63.3	0.0	4.3	31.6	56.1	100.0	100.0	100.0
35.0-44.9	204	59.3	54.3	64.4	0.0	9.9	26.0	53.2	100.0	100.0	100.0
45.0-54.9	198	62.2	57.5	66.8	9.5	17.4	34.0	60.7	100.0	100.0	100.0
55.0-64.9	129	58.4	51.8	65.0	0.0	3.3	20.1	56.3	100.0	100.0	100.0
65.0-74.9	231	61.0	56.2	65.7	0.0	9.5	29.3	61.0	100.0	100.0	100.0
75+	65	57.5	47.4	67.6	0.0	0.0	19.4	65.9	100.0	100.0	100.0
Men:											
19.0-34.9	244	53.7	49.6	57.8	0.0	9.0	27.2	49.9	85.5	100.0	100.0
35.0-44.9	229	59.8	55.8	63.8	12.4	19.8	34.9	60.1	100.0	100.0	100.0
45.0-54.9	164	59.9	55.3	64.5	13.4	18.0	38.6	57.7	86.5	100.0	100.0
55.0-64.9	107	62.3	56.1	68.4	6.6	19.2	32.3	61.9	100.0	100.0	100.0
65.0-74.9	201	61.1	56.7	65.4	6.4	19.5	37.6	59.1	100.0	100.0	100.0

75+	36	60.4	48.2	72.5	0.0	1.0	34.0	62.5	100.0	100.0	100.0
Total:											
19.0-34.9	484	56.2	53.1	59.3	0.0	6.2	28.9	52.6	100.0	100.0	100.0
35.0-44.9	433	59.6	56.4	62.8	0.7	13.3	30.6	57.9	100.0	100.0	100.0
45.0-54.9	362	61.2	57.9	64.4	12.5	17.7	35.9	59.1	100.0	100.0	100.0
55.0-64.9	236	60.2	55.6	64.7	0.0	7.8	28.1	61.3	100.0	100.0	100.0
65.0-74.9	432	61.0	57.8	64.3	0.0	13.0	33.9	60.4	100.0	100.0	100.0
75+	101	58.5	50.8	66.2	0.0	0.0	25.9	64.3	100.0	100.0	100.0

CI – confidence interval
